# The Impact of Race in Male Breast Cancer Treatment and Outcome in the United States: A Population-Based Analysis of 4,279 Patients

**DOI:** 10.1155/2014/685842

**Published:** 2014-10-02

**Authors:** Jacob Y. Shin, Lisa A. Kachnic, Ariel E. Hirsch

**Affiliations:** Department of Radiation Oncology, Boston Medical Center and Boston University School of Medicine, 830 Harrison Avenue, Moakley Building LL 100, Boston, MA 02118, USA

## Abstract

The purpose of this study is to compare the racial differences in treatment and overall survival (OS) of male breast cancer (MBC) patients. Data were extracted from the NCI SEER database that included population-based registries from 1988 to 2010 and analyzed using SPSS 20.0. 4,279 MBC patients were identified. 3,266 (76.3%) patients were White, 552 (12.9%) Black, 246 (5.7%) Hispanic, and 215 (5.0%) Asian. Black patients were more likely to be diagnosed at younger age (*P* < 0.001), have advanced stage disease (*P* = 0.001), and be unmarried (*P* < 0.001) and less likely to undergo lymph node dissection (*P* = 0.006). When stratified by stage, there was no difference in receipt of primary treatment by race. The 5-year OS for White, Black, Hispanic, and Asian races was 73.8%, 66.3%, 74.0%, and 85.3% (*P* < 0.001). This significant worse 5-year OS for Blacks persisted regardless of age, stage II or III disease, and grade 2 or 3 disease. On multivariate analysis, Black race was a significant independent prognostic factor for worse OS. Blacks were less likely to receive lymph node dissection of which patients may derive benefit, though we did not observe receipt of primary treatment, after stratifying for disease stage, to be an underlying factor contributing to racial outcome differences.

## 1. Introduction

In 2014, there will be an estimated 2,240 new cases of male breast cancer (MBC) in the United States, accounting for approximately 1% of all breast cancers annually [1]. It is estimated that 410 of these cases will end in death [1]. Though a rare disease, a previous population-based analysis has shown that the incidence has significantly increased from 0.86 to 1.08 per 100,000 population from 1973 to 1998, with incidence reaching as high as 1.24 per 100,000 man-years in 2000 [2, 3]. Mortality and survival rates have been shown to improve significantly over time, but to a significantly lesser extent in males compared to their female counterparts [3]. Age, tumor size, and nodal status have been shown to impact outcome in MBC patients [3–6].

However, the impact of race on MBC survival is still somewhat conflicting [7–11], which is in contrast to that seen in female breast cancer in which Blacks have been shown to have worse survival than Whites [12, 13]. Patients with MBC are more likely to be Black compared to their female counterparts (23% versus 16%, *P* = 0.013) [14], and the incidence of MBC is approximately 1.6 times higher in Black men when compared to Whites (1.8 versus 1.1 per 100,00 man-years) [9]. Thus, further understanding of the differences in presentation, treatment, and outcome by race in MBC patients is warranted.

In this population-based analysis, we sought to update our understanding of the impact of race on survival in MBC, analyzing differences in demographics, clinicopathologic characteristics, and treatment types to further define relationships between race and outcome.

## 2. Methods and Materials

### 2.1. Patient Population

Data was extracted from the Surveillance, Epidemiology, and End Results (SEER) database of the United States National Cancer Institute. It includes eighteen population-based registries representing approximately 28% of the U.S. population [15]. Our study population consists of 4,279 male patients whose first, primary malignancy was diagnosed as breast cancer between 1988 and 2010. Patients younger than 18 years, with unknown disease stage, or whose primary treatment type was unknown were excluded. Additionally, if race was other than White, Black, Hispanic, or Asian, further exclusions were made.

### 2.2. Statistical Analysis

SPSS 20.0 (Armonk, NY: IBM Corp.) was used to perform all data analyses. Demographic, clinicopathologic, and treatment data by race including proportional differences in age at diagnosis, year of diagnosis, primary treatment, stage of disease, tumor grade, and marital status were compared using the Chi-square test. Kaplan-Meier estimates were used to analyze overall survival (OS) and disease-specific survival (DSS). The OS endpoint was defined as time to death from the date of diagnosis of breast cancer. The DSS endpoint was defined as time to death with cause of death being breast cancer, from the date of diagnosis of breast cancer. The Cox regression multivariate analysis was used to identify independent predictors for OS. A two-sided *P* value <0.05 was considered statistically significant.

## 3. Results

### 3.1. Patient and Treatment Characteristics

4,279 MBC patients were identified and the patient and treatment characteristics are displayed in Table 1. The median age of the study population was 65 years (range: 23–99). Racial demographics included 3,266 (76.3%) Whites, 552 (12.9%) Blacks, 246 (5.7%) Hispanics, and 215 (5.0%) Asians. The majority of patients, 2,919 (68.2%), were married. Pathology included 3,552 (83.0%) patients with invasive ductal carcinoma, 145 (3.4%) lobular carcinoma, 73 (1.7%) mucinous carcinoma, 61 (1.4%) papillary carcinoma, 9 (0.2%) medullary carcinoma, and 439 (10.3%) ductal carcinoma in situ (DCIS). According to the AJCC 2010 staging system, 1,314 (30.7%) patients were stage I, 1,628 (38.0%) stage II, 668 (15.6%) stage III, and 230 (5.4%) stage IV. The majority of patients, 2,738 (64.0%), did not have nodal metastases.

Modified radical mastectomy was performed in 2,424 (56.6%) patients, while 1,027 (24.0%) underwent simple mastectomy, 511 (11.9%) partial mastectomy, 166 (3.9%) no treatment, 59 (1.4%) primary radiotherapy (RT), 41 (1.0%) radical mastectomy, and 22 (0.5%) nipple-sparing mastectomy. Lymph node dissection was performed in 3,583 (83.7%) patients, and 932 (21.8%) received adjuvant RT.

Blacks were more likely to be diagnosed at a younger age when compared with Whites, Hispanics, and Asians (61.2% versus 43.8% versus 59.8% versus 54.0%; *P* < 0.001). They were also less likely to be married compared to the other races (52.2% versus 70.2% versus 67.1% versus 80.5%; *P* < 0.001). Twenty-one percent of Blacks did not undergo lymph node dissection compared to 15.3% in Whites, 18.7% in Hispanics, and 15.8% in Asians (*P* = 0.006). A significantly higher proportion of Blacks were more likely to be diagnosed with advanced stage disease compared to the other races (stage III: 18.7% versus 15.2% versus 15.9% versus 14.0%, stage IV: 9.1% versus 4.8% versus 5.3% versus 4.2%; *P* = 0.001). Blacks were also less likely to have estrogen receptor (ER) and progesterone receptor (PR) positive disease when compared to Whites, Hispanics, and Asians (56.0% versus 64.8% versus 56.9% versus 67.0%; *P* < 0.001). When removing DCIS patients, significant racial differences were still observed when comparing by age (*P* < 0.001), marital status (*P* < 0.001), lymph node dissection (*P* = 0.001), stage of disease (*P* = 0.001), and ER/PR status (*P* < 0.001).

When stratified by stage, there was no difference in receipt of primary treatment with respect to race (Table 2).

### 3.2. Survival Outcome

The median follow-up for the whole patient cohort was 54 months (range: 0–275). The 5-year overall survival rate (OS) for the study population was 73.4%. Patients ≥65 years had significantly worse 5-year OS when compared with those <65 years (64.5% versus 83.2%; *P* < 0.001). There was a significant OS improvement seen over the time periods 1988–1993, 1994–1999, and 2000–2005 (68.9% versus 72.0% versus 75.2%; *P* = 0.018). Those who were married had significantly higher 5-year OS when compared with those divorced, single, widowed, or separated (77.5% versus 68.9% versus 68.7% versus 46.0%; *P* < 0.001) (Table 3).

Patients who did not receive any treatment (45.9%) or those who received primary RT (31.8%) had the worst 5-year OS when compared to any type of mastectomy (*P* < 0.001). There was a significant survival benefit for those who underwent lymph node dissection compared to those who did not (76.0% versus 61.0%; *P* < 0.001). No survival difference was seen between those who did or did not receive adjuvant RT (*P* = 0.738).

Patients with nodal metastases had significantly worse 5-year OS than those who did not (66.8% versus 77.0%; *P* < 0.001). Five-year OS by disease stage was as follows: 91.3% for DCIS (stage 0), 83.8% for stage I, 74.2% stage II, 55.5% stage III, and 19.4% stage IV (*P* < 0.001). Five-year OS for tumor grades 1, 2, and 3 was 84.7%, 76.5%, and 65.2%, respectively (*P* < 0.001).

**Figure 1:**
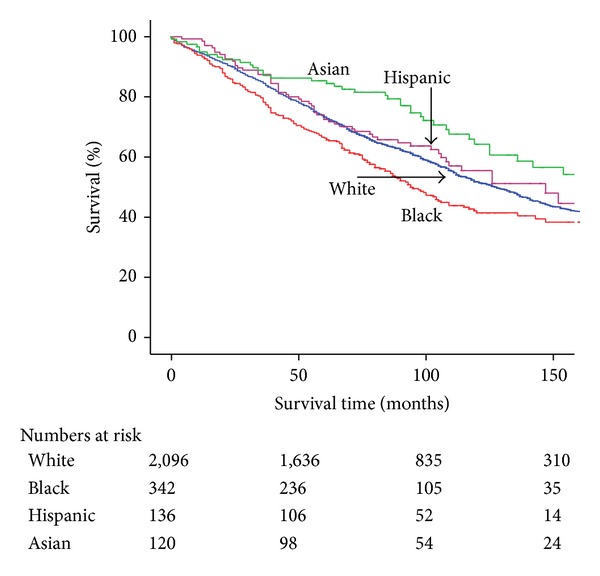
Kaplan-Meier overall survival by race.

**Figure 2:**
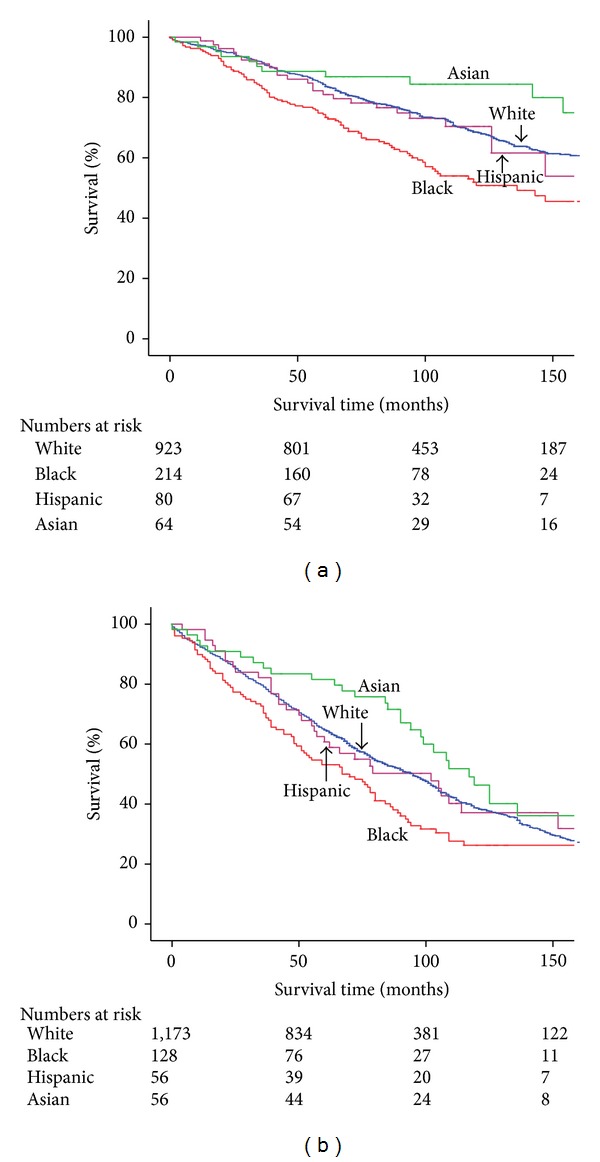
(a) Kaplan-Meier overall survival by race: <65 years. (b) Kaplan-Meier overall survival by race: ≥65 years.

Blacks had significantly worse 5-year OS when compared with Whites, Hispanics, and Asians (66.3% versus 73.8% versus 74.0% versus 85.3%; *P* < 0.001) (Figure 1). Blacks also had worse 5-year OS in those <65 years (74.3% versus 84.8% versus 80.9% versus 88.6%; *P* < 0.001) and in those ≥65 years (53.1% versus 65.1% versus 60.7% versus 81.6%; *P* = 0.011) compared to the other races (Figures 2(a) and 2(b)). In those with invasive ductal carcinoma, 5-year OS was significantly worse for Blacks (62.1% versus 71.2% versus 69.2% versus 83.5%; *P* < 0.001). Of patients with ER+/PR+ status, 5-year OS was 66.1% for Blacks versus 73.2% for Whites, 74.3% for Hispanics, and 89.6% for Asians (*P* = 0.006).

Blacks had significantly worse 5-year OS compared to Whites, Hispanics, and Asians in those who underwent modified radical mastectomy (64.9% versus 74.9% versus 71.6% versus 87.2%; *P* = 0.002), lymph node dissection (68.3% versus 76.3% versus 75.7% versus 89.6%; *P* < 0.001), and adjuvant RT (64.4% versus 75.0% versus 67.9% versus 95.7%; *P* = 0.003). In those with nodal metastases, Blacks also had significantly worse 5-year OS compared to the other races (53.3% versus 67.7% versus 69.6% versus 85.4%; *P* < 0.001). Of stage II patients, 5-year OS was 67.0% in Blacks, 74.2% in Whites, 79.5% in Hispanics, and 88.8% in Asians (*P* = 0.005). Five-year OS was also significantly worse in Blacks compared to the other races among stage III patients (37.0% versus 57.8% versus 64.5% versus 61.5%; *P* = 0.005). Finally, this survival disadvantage for Blacks compared to Whites, Hispanics, and Asians persisted in those with grade 2 (67.8% versus 76.9% versus 74.2% versus 94.1%; *P* = 0.005) or grade 3 (57.4% versus 65.7% versus 65.1% versus 77.1%; *P* = 0.014) disease. When removing DCIS patients, significant racial survival differences were still observed of those <65 years (*P* < 0.001) and ≥65 years of age (*P* = 0.002), married (*P* = 0.002), with ER+/PR+ disease (*P* = 0.001), undergoing modified radical mastectomy (*P* < 0.001), undergoing lymph node dissection (*P* < 0.001), undergoing adjuvant RT (*P* = 0.003), with nodal metastases (*P* < 0.001), with stage II (*P* = 0.005) or stage III (*P* = 0.005) disease, and with grade 2 (*P* = 0.005) or grade 3 (*P* = 0.016) disease.

Significant survival differences were also observed when DSS was analyzed (Table 4). Overall, Black patients had worse 5-year DSS when compared to Whites, Hispanics, and Asians (66.3% versus 73.8% versus 74.0% versus 85.3%; *P* < 0.001). Significantly worse survival for Black patients was also seen in those <65 years of age (*P* < 0.001), ≥65 years (*P* = 0.011), with invasive ductal carcinoma (*P* < 0.001), with ER+/PR+ disease (*P* = 0.006), undergoing modified radical mastectomy (*P* = 0.002), receiving lymph node dissection (*P* < 0.001), undergoing adjuvant radiotherapy (*P* = 0.003), with nodal metastasis (*P* < 0.001), with stage II (*P* = 0.005) and stage III (*P* = 0.005) disease, and with grade II (*P* = 0.005) and grade III (*P* = 0.014) disease when compared to the other races.

### 3.3. Multivariate Analysis

On Cox regression multivariate analysis, Black race (*P* < 0.001), older age at diagnosis (*P* < 0.001), more advanced T stage (*P* < 0.001), more advanced stage of disease (*P* < 0.001), and higher tumor grade (*P* < 0.001) were independent prognostic factors for worse OS. Later year of diagnosis (*P* = 0.024) and undergoing lymph node dissection (*P* < 0.001) were significant predictors for better OS. Black race remained an independent prognostic factor for worse OS even after adjusting for the other stated factors (Table 5).

## 4. Discussion

### 4.1. Impact of Race

Our current study on male adults across the U.S. diagnosed from 1988 to 2010 with MBC demonstrated that race remains a significant prognostic factor. Specifically, Blacks were shown to have significantly worse OS compared to other races, which may be due in part to more advanced disease presentation. Interestingly, we found that there was no significant difference with regard to receipt of cancer-directed primary treatment by race after stratifying by stage of disease. However, Blacks were less likely to undergo lymph node dissection when compared to the other races.

Similar to our findings, O'Malley et al. showed in a retrospective study on 1,979 MBC patients that 5-year survival for Black MBC patients was significantly worse compared to other races [11]. In those 65 years or older with MBC, Crew et al. also showed that Black race was associated with increased cancer-specific mortality when compared to White men (HR = 3.29, 95% CI = 1.10–9.86) [10]. Our report confirmed this survival disparity between races in MBC and revealed that it persists even after adjusting for age at diagnosis, year of diagnosis, primary treatment, stage of disease, and tumor grade. In contrast, a retrospective study on MBC cases in the Detroit metropolitan area by Simon et al. did not find race to be a predictor for survival [7]. These authors showed that Blacks (1.42, 95% CI = 1.07–1.77) had higher age-adjusted incidence rates of MBC per 100,000 compared to Whites (0.88, 95% CI = 0.86–1.00), but that race was not a significant prognostic factor for survival (relative risk = 1.18, 95% CI = 0.77–1.82). Another study on six hundred patients from the California Cancer Registry did not observe any significant differences in outcome by race when comparing Blacks to Whites (HR = 1.32, 95% CI = 0.75–2.33) after adjusting for age at diagnosis and stage of disease [8].

Several possibilities exist for the basis of our finding of racial disparity in OS. Previous studies have shown that Blacks have higher incidence rates of large tumor size, nodal metastases, high tumor grade, and negative hormone receptor expression compared to Whites [8, 9, 11]. These results are in concordance with our findings of more advanced disease presentation in Blacks compared to other racial groups. There also exists the prospect that Blacks have suboptimal access to specialist care. Crew et al. found that, in those 65 years of age or older, Black men were about half as likely to be seen by a medical oncologist for initial consult and receive adjuvant chemotherapy when compared to Whites, though this did not reach statistical significance [10]. A retrospective study among 9,630 women, 66 years of age or older and with early-stage breast cancer, showed that those who saw a medical oncologist before surgery were more likely to undergo definitive surgery and axillary lymph node dissection [16]. Speculatively, this may have contributed to the lower rates of lymph node dissection seen in Blacks in our study. However, the SEER database lacks information on provider specialty, and thus, we are unable to determine if this had an effect in our analysis.

Differences in receipt of treatment between races have also been investigated in previous studies [7]. The majority of patients undergo modified radical mastectomy [11], and previous studies have shown no difference in local recurrence or survival rates in comparison to radical mastectomy [17]. Additionally, a previous retrospective study by Fogh et al. showed breast conservation surgery to have comparable rates of local control, disease-free survival, and overall survival with modified radical mastectomy or total simple mastectomy [18]. Simon et al. found no significant differences in the surgical treatment or administration of RT between Black and White males [7]. Similarly, we found that there was no significant difference between racial groups with regard to receipt of primary cancer-directed treatment when stratified by stage of disease. However, we did find that Blacks were less likely to undergo lymph node dissection. In a multi-institutional retrospective study on 397 men with nonmetastatic breast cancer, Cutuli et al. showed that men who undergo nodal dissection may derive some benefit in outcome. These authors showed that regional nodal recurrence occurred in only 1.2% of patients who underwent axillary dissection compared to 13% in those without dissection (*P* < 0.001) [19]. However, the SEER database does not record disease recurrence. Finally, we did not note a difference in receipt of adjuvant RT between racial groups. Though there are still limited data from small retrospective studies regarding the indications for postmastectomy RT in MBC and its impact on outcome [20–22], a prior retrospective study by Fogh et al., on forty-two male nonmetastatic ER+/PR+ breast cancer patients, showed adjuvant RT and tamoxifen to be superior in terms of overall survival to adjuvant tamoxifen alone, adjuvant RT alone, and no adjuvant therapy, suggesting a possible role for adjuvant RT in addition to tamoxifen in ER+/PR+ MBC [23]. Thus, further studies are warranted to justify its use for this patient population.

In our analysis, age was divided by the median age of 65 years, and younger age was defined as those younger than 65 years of age. Black males were more likely to be diagnosed with breast cancer at a younger age compared to other races. Previous studies have shown that, in women younger than 35 years of age, Black patients were at increased risk for breast cancer compared to other races and that they have an increased mortality risk when compared with White women of the same age [24, 25]. Black MBC patients in our study were also less likely to be married compared to other races. Unmarried patients in our analysis were shown to have worse outcome, and there certainly exists a possibility of a relation between the respective associations of younger age and being nonmarried for Black patients. Interestingly, a recent report by the U.S. Bureau of Labor Statistics has shown that Blacks married at a later age and at lower rates when compared with Whites [26].

Black MBC patients were significantly more likely to have ER−/PR− disease (5.3% versus 4.0%, *P* < 0.001) and less likely to have ER+/PR+ disease (56.0% versus 64.8%, *P* < 0.001) when compared to White patients. These findings are in concordance with previous studies showing an increased association of triple-negative breast cancer in Black female breast cancer patients when compared to other races [27]. A higher proportion of ER−/PR− disease observed in Black MBC patients compared to Whites may be a contributing factor to their poorer outcome. Prior investigations have shown triple-negative breast cancer to be associated with poorer outcome [28, 29]; however, a limitation of the SEER database is that HER2 status, which has been associated with outcome in female breast cancer patients, is not recorded [30].

An interesting finding from our study is the significantly better OS observed in Asian MBC patients compared to the other races of those ≥65 years (*P* = 0.011), married (*P* = 0.037), with invasive ductal carcinoma (*P* < 0.001), with ER+/PR+ disease (*P* = 0.006), receiving modified radical mastectomy (*P* = 0.002), undergoing lymph node dissection (*P* < 0.001), with nodal metastasis (*P* < 0.001), and with grade 2-3 disease (*P* = 0.005, *P* = 0.014). A previous study on prostate cancer patients showed that Japanese men had significantly better survival than White patients; however, the reasons were unclear [31]. In advanced stage, non-small cell lung cancer patients, Asians were found to have significantly higher survival rates and greater response rates to chemotherapy [32]. In a study on female breast cancer patients, Japanese women were also found to have significantly better survival compared to White patients, but there were no significant differences between Chinese, Filipino, and White women [33]. In our study, they were more likely to present with earlier stage disease (*P* = 0.001), and these results are similar to those seen in previous studies for other cancer sites [34]. Though Asian MBC patients make up only a small percentage of those with breast cancer, further studies are warranted to investigate possible etiologies for differences in outcome and the heterogeneity observed in this fast-growing population in the United States [35].

### 4.2. Prognostic Factors

A previous retrospective study on 2,537 MBC patients showed that age ≥65 years (HR = 1.59), tumor size 2–5 cm (HR = 1.40), and nodal metastases (HR = 1.50) were independent prognostic factors for worse survival [2]. In a single-institutional study on twenty-nine MBC patients, Moore et al. also showed that older age was a significant predictor for worse OS (*P* = 0.008) [4]. Indeed, we found that, in addition to Black race, older age at diagnosis, earlier year of diagnosis, not undergoing mastectomy, more advanced stage of disease, and higher tumor grade portended for worse OS on multivariate analysis.

The majority of new cancers and cancer deaths have been shown to occur in those 65 years of age or older [36], and our analysis showed that those >65 years of age had worse OS. Previous studies have shown older age to be associated with chronic illness and age-related health conditions which can have adverse impact on quality of life, rates of disability, and independent living [37, 38]. The preexisting health conditions of a patient may have an impact on clinical decision making and cancer treatment. Appropriate assessment and management of preexisting comorbidities can potentially provide benefit, enhance quality of life, and, subsequently, impact patient outcome.

Our study cohort, consisting of patients from 1988 to 2010, showed an improvement in 5-year OS over time. We observed a significant improvement in 5-year OS for all MBC patients over the time periods 1988–1993, 1994–1999, and 2000–2005 (68.9% versus 72.0% versus 75.2%; *P* = 0.018). Similarly, Anderson et al. showed that hazard ratios in MBC patients fell by 28% from the 1976–1985 time period to the 1996–2005 period (*P* = 0.03) [3]. Year of diagnosis was not shown to affect survival in a prior retrospective study; however, this report was limited to patients diagnosed in 1997 or earlier [11]. There exist several possible factors for our finding, including more advanced treatments and improved multidisciplinary approach to cancer care with time.

A report by Aizer et al. found that unmarried patients were at significant risk for suboptimal management, metastatic disease, and poor outcome in a study on over one million patients diagnosed with lung, breast, prostate, colorectal, liver/intrahepatic bile duct, pancreatic, non-Hodgkin lymphoma, head/neck, ovarian, or esophageal cancer [39]. On univariate analysis, our report on MBC showed that those who were married had the highest 5-year OS of any marital status subgroup. However, marital status was not found to be a significant prognostic factor in our multivariate analysis. Nonetheless, there is now awareness that those unmarried may have poorer outcomes and that social support services have the potential to have a positive impact on treatment adherence, response, and survival.

To our knowledge, this report is the largest comprehensive study on MBC to examine the impact of race on outcome, as well as prognostic factors for survival in these patients. The strength in our study lies in the large patient numbers from four racial groups, White, Black, Hispanic, and Asian, which allowed for statistical analysis powered for detection of differences in treatment and outcome and investigation of different prognostic factors in multivariate analysis. Prior studies examining race in MBC have been limited by either geographic distribution [7, 8], age at diagnosis [10], or analyzed much earlier time periods [11]. In contrast, this SEER population-based cancer analysis is broadly representative of the U.S. population, covering approximately 28 percent of the population, thus decreasing selection bias risks that may be associated with smaller analyses [15]. However, our current study also has several limitations including no record on centralized pathology review, margin status after primary surgery, adjuvant systemic therapy, disease recurrence, and information on risk factors for MBC such as first-degree relative with breast cancer, BRCA status, hormone levels, body mass index, and obesity [40, 41]. In addition, our analysis lacked information on cultural, socioeconomic, and behavioral factors which may have an effect on outcome. Thus, we cannot evaluate the extent to which these factors may contribute to racial disparities.

### 4.3. Conclusions

In summary, the results of this SEER analysis of 4,279 MBC patients showed that race, age at diagnosis, year of diagnosis, T stage, stage of disease, and tumor grade are independent predictors for survival. Blacks were found to have an overall survival disadvantage compared to Whites, Hispanics, and Asians, which may be in part due to differences in disease presentation. Our study did show that Blacks were less likely to receive lymph node dissection of which patients may derive benefit, though we did not observe receipt of primary treatment, after stratifying for stage of disease, to be an underlying factor contributing to this disparity in survival outcome. Further studies are warranted to investigate if racial disparities in MBC are associated with socioeconomic status, access to medical care, limiting medical comorbidities, and genetic and biologic etiologies.

## Figures and Tables

**Table 1:** Demographic and clinicopathologic characteristics by race.

	Total	White	Black	Hispanic	Asian	Chi-square *P* value
Overall	4,279	3,266	552	246	215	
Age						*P* < 0.001
Median (range)	65 (23–99)					
<65 years	2,032 (47.5%)	1,431 (43.8%)	338 (61.2%)	147 (59.8%)	116 (54.0%)	
≥65 years	2,247 (52.5%)	1,835 (56.2%)	214 (38.8%)	99 (40.2%)	99 (46.0%)	
Year of diagnosis						*P* = 0.012
1988–1993	457 (10.7%)	366 (11.2%)	48 (8.7%)	17 (6.9%)	26 (12.1%)	
1994–1999	665 (15.5%)	518 (15.9%)	81 (14.7%)	33 (13.4%)	33 (15.3%)	
2000–2005	1,572 (36.7%)	1,212 (37.1%)	213 (38.6%)	86 (35.0%)	61 (28.4%)	
2006–2010	1,585 (37.0%)	1,170 (35.8%)	210 (38.0%)	110 (44.7%)	95 (44.2%)	
Marital status						*P* < 0.001
Single	526 (12.3%)	339 (10.4%)	128 (23.2%)	42 (17.1%)	17 (7.9%)	
Married	2,919 (68.2%)	2,293 (70.2%)	288 (52.2%)	165 (67.1%)	173 (80.5%)	
Separated	35 (0.8%)	18 (0.6%)	12 (2.2%)	4 (1.6%)	1 (0.5%)	
Divorced	289 (6.8%)	213 (6.5%)	55 (10.0%)	15 (6.1%)	6 (2.8%)	
Widowed	321 (7.5%)	259 (7.9%)	39 (7.1%)	11 (4.5%)	12 (5.6%)	
Unknown	189 (4.4%)	144 (4.4%)	30 (5.4%)	9 (3.7%)	6 (2.8%)	
Histology						*P* = 0.003
In situ	439 (10.3%)	326 (10.0%)	58 (10.5%)	35 (14.2%)	20 (9.3%)	
Ductal	3,552 (83.0%)	2,736 (83.8%)	442 (80.1%)	194 (78.9%)	180 (83.7%)	
Papillary	61 (1.4%)	45 (1.4%)	11 (2.0%)	3 (1.2%)	2 (0.9%)	
Mucinous	73 (1.7%)	42 (1.3%)	22 (4.0%)	5 (2.0%)	4 (1.9%)	
Medullary	9 (0.2%)	6 (0.2%)	0 (0.0%)	1 (0.4%)	2 (0.9%)	
Lobular	145 (3.4%)	111 (3.4%)	19 (3.4%)	8 (3.3%)	7 (3.3%)	
ER/PR status						*P* < 0.001
ER−/PR−	187 (4.4%)	130 (4.0%)	29 (5.3%)	14 (5.7%)	14 (6.5%)	
ER+/PR−	383 (9.0%)	280 (8.6%)	70 (12.7%)	22 (8.9%)	11 (5.1%)	
ER+/PR+	2,709 (63.3%)	2,116 (64.8%)	309 (56.0%)	140 (56.9%)	144 (67.0%)	
Unknown	1,000 (23.4%)	740 (22.7%)	144 (26.1%)	70 (28.5%)	46 (21.4%)	
Treatment						*P* < 0.001
None	166 (3.9%)	101 (3.1%)	37 (6.7%)	13 (5.3%)	15 (7.0%)	
Primary RT	59 (1.4%)	43 (1.3%)	12 (2.2%)	4 (1.6%)	0 (0.0%)	
Partial mast.	511 (11.9%)	372 (11.4%)	68 (12.3%)	41 (16.7%)	30 (14.0%)	
Nipple-sparing mast.	22 (0.5%)	16 (0.5%)	5 (0.9%)	1 (0.4%)	0 (0.0%)	
Simple mast.	1,027 (24.0%)	809 (24.8%)	116 (21.0%)	51 (20.7%)	51 (23.7%)	
Modified rad. mast.	2,424 (56.6%)	1,869 (57.2%)	304 (55.1%)	135 (54.9%)	116 (54.0%)	
Rad. mast.	41 (1.0%)	31 (0.9%)	9 (1.6%)	1 (0.4%)	0 (0.0%)	
Surgery, NOS	29 (0.7%)	25 (0.8%)	1 (0.2%)	0 (0.0%)	3 (1.4%)	
LNDX						*P* = 0.006
No	696 (16.3%)	500 (15.3%)	116 (21.0%)	46 (18.7%)	34 (15.8%)	
Yes	3,583 (83.7%)	2,766 (84.7%)	436 (79.0%)	200 (81.3%)	181 (84.2%)	
Adjuvant RT						*P* = 0.588
No	3,288 (76.8%)	2,503 (76.6%)	419 (75.9%)	197 (80.1%)	169 (78.6%)	
Yes	932 (21.8%)	720 (22.0%)	121 (21.9%)	45 (18.3%)	46 (21.4%)	
Nodal metastases						*P* = 0.659
No	2,738 (64.0%)	2,093 (64.1%)	343 (62.1%)	164 (66.7%)	138 (64.2%)	
Yes	1,541 (36.0%)	1,173 (35.9%)	209 (37.9%)	82 (33.3%)	77 (35.8%)	
Stage						*P* = 0.001
0	439 (10.3%)	326 (10.0%)	58 (10.5%)	35 (14.2%)	20 (9.3%)	
I	1,314 (30.7%)	1,023 (31.3%)	142 (25.7%)	75 (30.5%)	74 (34.4%)	
II	1,628 (38.0%)	1,263 (38.7%)	199 (36.1%)	84 (34.1%)	82 (38.1%)	
III	668 (15.6%)	496 (15.2%)	103 (18.7%)	39 (15.9%)	30 (14.0%)	
IV	230 (5.4%)	158 (4.8%)	50 (9.1%)	13 (5.3%)	9 (4.2%)	
Grade						*P* = 0.414
1	466 (10.9%)	350 (10.7%)	59 (10.7%)	32 (13.0%)	25 (11.6%)	
2	1,859 (43.4%)	1,448 (44.3%)	232 (42.0%)	85 (34.6%)	94 (43.7%)	
3	1,374 (32.1%)	1,042 (31.9%)	176 (31.9%)	83 (33.7%)	73 (34.0%)	
Unknown	580 (13.6%)	426 (13.0%)	85 (15.4%)	46 (18.7%)	23 (10.7%)	

Mast = mastectomy; RT = radiotherapy; LNDX = lymph node dissection; ER = estrogen receptor; PR = progesterone receptor.

**Table 2:** Primary treatment by stage and race.

Stage	Total	White	Black	Hispanic	Asian	Chi-square *P* value
0						*P* = 0.357
None	37 (8.5%)	25 (7.7%)	4 (6.9%)	4 (11.4%)	4 (21.1%)	
RT alone	2 (0.5%)	2 (0.6%)	0 (0.0%)	0 (0.0%)	0 (0.0%)	
Partial mast.	112 (25.7%)	73 (22.6%)	19 (32.8%)	13 (37.1%)	7 (36.8%)	
Nipple-sparing mast.	8 (1.8%)	6 (1.9%)	2 (3.4%)	0 (0.0%)	0 (0.0%)	
Simple mast.	201 (46.2%)	158 (48.9%)	22 (37.9%)	14 (40.0%)	7 (36.8%)	
Modified rad. mast.	75 (17.2%)	59 (18.3%)	11 (19.0%)	4 (11.4%)	1 (5.3%)	
Rad. mast.	0 (0.0%)	0 (0.0%)	0 (0.0%)	0 (0.0%)	0 (0.0%)	
I						*P* = 0.886
None	22 (1.7%)	17 (1.7%)	2 (1.4%)	2 (2.7%)	1 (1.4%)	
Primary RT	4 (0.3%)	4 (0.4%)	0 (0.0%)	0 (0.0%)	0 (0.0%)	
Partial mast.	200 (15.3%)	150 (14.7%)	21 (14.8%)	18 (24.0%)	11 (15.1%)	
Nipple-sparing mast.	8 (0.6%)	5 (0.5%)	2 (1.4%)	1 (1.3%)	0 (0.0%)	
Simple mast.	366 (28.0%)	285 (28.0%)	43 (30.3%)	16 (21.3%)	22 (30.1%)	
Modified rad. mast.	700 (53.6%)	550 (54.1%)	73 (51.4%)	38 (50.7%)	39 (53.4%)	
Rad. mast.	7 (0.5%)	6 (0.6%)	1 (0.7%)	0 (0.0%)	0 (0.0%)	
II						*P* = 0.226
None	21 (1.3%)	12 (1.0%)	7 (3.5%)	0 (0.0%)	2 (2.5%)	
Primary RT	7 (0.4%)	5 (0.4%)	1 (0.5%)	1 (1.2%)	0 (0.0%)	
Partial mast.	149 (9.2%)	112 (9.0%)	18 (9.0%)	8 (9.5%)	11 (13.6%)	
Nipple-sparing mast.	5 (0.3%)	5 (0.4%)	0 (0.0%)	0 (0.0%)	0 (0.0%)	
Simple mast.	355 (22.0%)	286 (22.9%)	38 (19.1%)	16 (19.0%)	15 (18.5%)	
Modified rad. mast.	1,060 (65.6%)	819 (65.5%)	130 (65.3%)	58 (69.0%)	53 (65.4%)	
Rad. mast.	18 (1.1%)	12 (1.0%)	5 (2.5%)	1 (1.2%)	0 (0.0%)	
III						*P* = 0.402
None	26 (3.9%)	14 (2.8%)	9 (8.7%)	1 (2.6%)	2 (6.7%)	
Primary RT	3 (0.5%)	3 (0.6%)	0 (0.0%)	0 (0.0%)	0 (0.0%)	
Partial mast.	26 (3.9%)	20 (4.1%)	3 (2.9%)	2 (5.1%)	1 (3.3%)	
Nipple-sparing mast.	1 (0.2%)	0 (0.0%)	1 (1.0%)	0 (0.0%)	0 (0.0%)	
Simple mast.	75 (11.3%)	56 (11.4%)	9 (8.7%)	5 (12.8%)	5 (16.7%)	
Modified rad. mast.	521 (78.3%)	390 (79.1%)	78 (75.7%)	31 (79.5%)	22 (73.3%)	
Rad. mast.	13 (2.0%)	10 (2.0%)	3 (2.9%)	0 (0.0%)	0 (0.0%)	
IV						*P* = 0.116
None	60 (26.3%)	33 (21.0%)	15 (30.6%)	6 (46.2%)	6 (66.7%)	
Primary RT	43 (18.9%)	29 (18.5%)	11 (22.4%)	3 (23.1%)	0 (0.0%)	
Partial mast.	24 (10.5%)	17 (10.8%)	7 (14.3%)	0 (0.0%)	0 (0.0%)	
Nipple-sparing mast.	0 (0.0%)	0 (0.0%)	0 (0.0%)	0 (0.0%)	0 (0.0%)	
Simple mast.	30 (13.2%)	24 (15.3%)	4 (8.2%)	0 (0.0%)	2 (22.2%)	
Modified rad. mast.	68 (29.8%)	51 (32.5%)	12 (24.5%)	4 (30.8%)	1 (11.1%)	
Rad. mast.	3 (1.3%)	3 (1.9%)	0 (0.0%)	0 (0.0%)	0 (0.0%)	

Mast = mastectomy; RT = radiotherapy.

**Table 3:** Five-year Kaplan-Meier overall survival by race.

	Total	White	Black	Hispanic	Asian	Log-rank *P* value
Overall	73.4% (±0.9)	73.8% (±1.2)	66.3% (±2.6)	74.0% (±3.8)	85.3% (±3.3)	*P* < 0.001
Age						*P* < 0.001
<65 years	83.2% (±1.1)	84.8% (±1.2)	74.3% (±3.0)	80.9% (±4.4)	88.6% (±4.0)	*P* < 0.001
≥65 years	64.5% (±1.3)	65.1% (±1.4)	53.1% (±4.4)	60.7% (±6.5)	81.6% (±5.3)	*P* = 0.011
Year of diagnosis						*P* = 0.018
1988–1993	68.9% (±2.2)	69.1% (±2.4)	60.4% (±7.1)	64.7% (±11.6)	80.8% (±7.7)	*P* = 0.549
1994–1999	72.0% (±1.7)	74.5% (±1.9)	55.6% (±5.5)	57.6% (±8.6)	87.5% (±5.8)	*P* < 0.001
2000–2005	75.2% (±1.1)	74.8% (±1.3)	71.8% (±3.1)	82.4% (±4.1)	86.2% (±4.5)	*P* = 0.031
Marital status						*P* < 0.001
Single	68.7% (±2.6)	69.8% (±3.1)	58.5% (±5.7)	76.6% (±8.4)	85.7% (±13.2)	*P* = 0.144
Married	77.5% (±1.0)	77.6% (±1.1)	73.7% (±3.3)	72.8% (±4.7)	87.4% (±3.4)	*P* = 0.037
Separated	46.0% (±10.2)	41.7% (±14.2)	44.4% (±16.6)	50.0% (±35.4)	—	*P* = 0.665
Divorced	68.9% (±3.4)	68.3% (±4.0)	72.7% (±7.8)	77.8% (±13.9)	33.3% (±27.2)	*P* = 0.528
Widowed	51.9% (±3.5)	53.1% (±3.9)	33.3% (±9.6)	71.4% (±17.1)	71.4% (±17.1)	*P* = 0.174
Unknown	66.7% (±4.9)	66.7% (±5.6)	65.7% (±12.5)	33.3% (±27.2)	100.0% (±0.0)	*P* = 0.694
Histology						*P* < 0.001
In situ	91.3% (±1.6)	90.0% (±2.0)	94.6% (±3.7)	94.1% (±5.7)	100.0% (±0.0)	*P* = 0.543
Ductal	70.6% (±1.0)	71.2% (±1.1)	62.1% (±3.0)	69.2% (±4.5)	83.5% (±3.9)	*P* < 0.001
Papillary	75.6% (±6.4)	75.0% (±7.7)	88.9% (±10.5)	50.0% (±35.4)	50.0% (±35.4)	*P* = 0.457
Mucinous	81.1% (±5.4)	87.5% (±5.8)	62.5% (±12.1)	100.0% (±0.0)	—	*P* = 0.034
Medullary	100.0% (±0.0)	100.0% (±0.0)	—	—	100.0% (±0.0)	—
Lobular	75.1% (±5.0)	75.2% (±5.6)	57.1% (±18.7)	50.0% (±25.0)	80.0% (±17.9)	*P* = 0.352
ER/PR status						*P* = 0.057
ER−/PR−	71.8% (±4.0)	71.8% (±4.9)	70.8% (±9.3)	57.1% (±18.7)	87.5% (±11.7)	*P* = 0.175
ER+/PR−	65.5% (±3.0)	67.8% (±3.4)	57.7% (±8.1)	55.6% (±16.6)	57.1% (±18.7)	*P* = 0.487
ER+/PR+	73.3% (±1.2)	73.2% (±1.3)	66.1% (±3.9)	74.3% (±5.2)	89.6% (±3.7)	*P* = 0.006
Unknown	76.1% (±1.5)	76.9% (±1.7)	68.4% (±4.1)	79.4% (±5.8)	81.9% (±6.7)	*P* = 0.045
Treatment						*P* < 0.001
None	45.9% (±5.1)	50.0% (±6.4)	33.3% (±10.3)	60.0% (±21.9)	35.7% (±19.8)	*P* = 0.329
Primary RT	31.8% (±7.0)	30.3% (±8.0)	42.9% (±18.7)	25.0% (±21.7)	—	*P* = 0.904
Partial mast.	80.0% (±2.6)	78.1% (±3.1)	78.8% (±7.1)	80.8% (±12.2)	100.0% (±0.0)	*P* = 0.271
Nipple-sparing mast.	75.0% (±10.8)	91.7% (±8.0)	25.0% (±21.7)	—	—	*P* = 0.004
Simple mast.	77.1% (±1.9)	75.5% (±2.2)	82.6% (±5.3)	88.5% (±6.3)	79.6% (±9.1)	*P* = 0.294
Modified rad. mast.	74.0% (±1.0)	74.9% (±1.2)	64.9% (±3.3)	71.6% (±4.8)	87.2% (±4.0)	*P* = 0.002
Rad. mast.	59.3% (±9.5)	52.6% (±11.5)	71.4% (±17.1)	—	—	*P* = 0.321
Surgery, NOS	80.0% (±10.3)	75.0% (±12.5)	100.0% (±0.0)	—	0.0% (±0.0)	*P* = 0.727
LNDX						*P* < 0.001
No	61.0% (±2.3)	60.9% (±2.6)	58.6% (±5.9)	66.2% (±9.8)	65.3% (±10.7)	*P* = 0.592
Yes	76.0% (±0.9)	76.3% (±1.0)	68.3% (±2.8)	75.7% (±4.1)	89.6% (±3.1)	*P* < 0.001
Adjuvant RT						*P* = 0.738
No	73.9% (±1.0)	74.3% (±1.1)	67.5% (±2.9)	75.6% (±4.2)	82.7% (±3.9)	*P* = 0.033
Yes	74.7% (±1.8)	75.0% (±2.1)	64.4% (±5.7)	67.9% (±8.8)	95.7% (±4.3)	*P* = 0.003
Nodal metastases						*P* < 0.001
No	77.0% (±1.0)	77.1% (±1.1)	74.1% (±3.0)	76.3% (±4.5)	85.3% (±4.1)	*P* = 0.101
Yes	66.8% (±1.5)	67.7% (±1.7)	53.3% (±4.4)	69.6% (±6.8)	85.4% (±5.5)	*P* < 0.001
Stage						*P* < 0.001
0	91.3% (±1.6)	90.0% (±2.0)	94.6% (±3.7)	94.1% (±5.7)	100.0% (±0.0)	*P* = 0.543
I	83.8% (±1.3)	83.5% (±1.5)	85.3% (±3.8)	76.9% (±6.7)	92.3% (±4.3)	*P* = 0.246
II	74.2% (±1.4)	74.2% (±1.6)	67.0% (±4.3)	79.5% (±6.1)	88.8% (±4.7)	*P* = 0.005
III	55.5% (±2.4)	57.8% (±2.8)	37.0% (±6.0)	64.5% (±9.0)	61.5% (±13.5)	*P* = 0.005
IV	19.4% (±3.4)	17.5% (±3.9)	30.8% (±9.1)	14.3% (±13.2)	—	*P* = 0.421
Grade						*P* < 0.001
1	84.7% (±2.1)	85.1% (±2.4)	76.5% (±7.3)	91.7% (±8.0)	92.9% (±6.9)	*P* = 0.390
2	76.5% (±1.3)	76.9% (±1.4)	67.8% (±4.1)	74.2% (±6.0)	94.1% (±3.3)	*P* = 0.005
3	65.2% (±1.6)	65.7% (±1.8)	57.4% (±4.8)	65.1% (±7.3)	77.1% (±7.1)	*P* = 0.014
Unknown	73.9% (±2.1)	73.9% (±2.4)	72.5% (±5.4)	72.5% (±8.9)	70.6% (±11.1)	*P* = 0.899

Mast = mastectomy; RT = radiotherapy; LNDX = lymph node dissection; ER = estrogen receptor; PR = progesterone receptor.

**Table 4:** Five-year Kaplan-Meier disease-specific survival by race.

	Total	White	Black	Hispanic	Asian	Log-rank *P* value
Overall	87.5% (±0.7)	88.6% (±0.7)	80.0% (±2.3)	86.6% (±3.0)	90.2% (±2.8)	*P* < 0.001
Age						*P* = 0.970
<65 years	88.9% (±0.9)	90.0% (±1.0)	84.7% (±2.6)	85.6% (±4.0)	88.6% (±4.0)	*P* = 0.002
≥65 years	86.2% (±1.0)	87.4% (±1.0)	71.6% (±4.3)	83.6% (±5.4)	92.1% (±3.8)	*P* < 0.001
Year of diagnosis						*P* = 0.215
1988–1993	86.6% (±1.7)	87.1% (±1.9)	81.4% (±6.0)	87.5% (±8.3)	88.0% (±6.5)	*P* = 0.683
1994–1999	86.8% (±1.4)	90.5% (±1.3)	66.8% (±5.6)	74.8% (±8.3)	87.5% (±5.8)	*P* < 0.001
2000–2005	88.1% (±0.9)	88.3% (±1.0)	84.6% (±2.6)	90.4% (±3.2)	92.8% (±3.5)	*P* = 0.056
Marital status						*P* < 0.001
Single	80.9% (±2.3)	82.7% (±2.7)	74.3% (±5.4)	83.5% (±7.6)	85.7% (±13.2)	*P* = 0.546
Married	90.2% (±0.7)	90.9% (±0.8)	85.3% (±2.7)	84.1% (±4.0)	92.4% (±2.8)	*P* = 0.005
Separated	54.5% (±10.7)	61.1% (±15.4)	44.4% (±16.6)	50.0% (±35.4)	—	*P* = 0.475
Divorced	81.8% (±3.0)	82.6% (±3.4)	81.1% (±7.0)	88.9% (±10.5)	33.3% (±27.2)	*P* = 0.086
Widowed	81.9% (±3.0)	83.4% (±3.2)	64.7% (±11.1)	100.0% (±0.0)	85.7% (±13.2)	*P* = 0.046
Unknown	85.3% (±3.9)	84.1% (±4.6)	85.7% (±9.4)	—	100.0% (±0.0)	*P* = 0.351
Histology						*P* < 0.001
In situ	99.3% (±0.5)	99.1% (±0.6)	100.0% (±0.0)	100.0% (±0.0)	100.0% (±0.0)	*P* = 0.874
Ductal	85.5% (±0.8)	86.8% (±0.9)	75.9% (±2.8)	82.8% (±3.8)	89.7% (±3.3)	*P* < 0.001
Papillary	92.9% (±4.0)	93.3% (±4.6)	100.0% (±0.0)	—	50.0% (±35.4)	*P* = 0.136
Mucinous	94.3% (±3.2)	96.9% (±3.1)	87.5% (±8.3)	100.0% (±0.0)	—	*P* = 0.744
Medullary	100.0% (±0.0)	100.0% (±0.0)	—	—	100.0% (±0.0)	—
Lobular	88.9% (±3.7)	89.4% (±4.1)	80.0% (±17.9)	50.0% (±25.0)	80.0% (±17.9)	*P* = 0.167
ER/PR status						*P* < 0.001
ER−/PR−	85.0% (±3.4)	83.6% (±4.2)	89.7% (±6.9)	71.4% (±17.1)	100.0% (±0.0)	*P* = 0.359
ER+/PR−	77.6% (±2.8)	80.7% (±3.0)	71.4% (±7.7)	55.6% (±16.6)	57.1% (±18.7)	*P* = 0.082
ER+/PR+	88.0% (±0.9)	88.7% (±1.0)	78.8% (±3.5)	89.1% (±3.9)	95.3% (±2.6)	*P* < 0.001
Unknown	89.7% (±1.1)	91.4% (±1.2)	82.2% (±3.5)	91.2% (±4.2)	84.8% (±6.3)	*P* = 0.008
Treatment						*P* < 0.001
None	61.4% (±5.4)	68.2% (±6.5)	42.9% (±11.4)	80.0% (±17.9)	41.7% (±22.2)	*P* = 0.131
Primary RT	40.2% (±7.9)	36.2% (±9.0)	53.6% (±20.1)	50.0% (±25.0)	—	*P* = 0.823
Partial mast.	92.8% (±1.7)	91.4% (±2.1)	93.8% (±4.3)	100.0% (±0.0)	100.0% (±0.0)	*P* = 0.673
Nipple-sparing mast.	100.0% (±0.0)	100.0% (±0.0)	—	—	—	—
Simple mast.	92.0% (±1.3)	92.5% (±1.4)	87.9% (±4.6)	95.8% (±4.1)	89.2% (±7.2)	*P* = 0.346
Modified rad. mast.	88.1% (±0.8)	89.4% (±0.9)	80.1% (±2.9)	84.2% (±4.0)	91.2% (±3.4)	*P* < 0.001
Rad. mast.	72.6% (±8.8)	72.2% (±10.6)	71.4% (±17.1)	—	—	*P* = 0.736
Surgery, NOS	85.7% (±9.4)	81.8% (±11.6)	—	—	81.8% (±11.6)	*P* = 0.711
LNDX						*P* < 0.001
No	78.7% (±2.0)	79.7% (±2.3)	69.6% (±5.7)	91.0% (±6.1)	78.0% (±9.8)	*P* = 0.045
Yes	89.3% (±0.7)	90.4% (±0.7)	82.6% (±2.4)	85.7% (±3.4)	92.6% (±2.7)	*P* < 0.001
Adjuvant RT						*P* < 0.001
No	89.2% (±0.7)	90.7% (±0.8)	80.9% (±2.5)	88.4% (±3.3)	87.7% (±3.5)	*P* < 0.001
Yes	85.2% (±1.6)	85.0% (±1.8)	79.1% (±5.2)	85.2% (±6.8)	100.0% (±0.0)	*P* = 0.019
Nodal metastases						*P* < 0.001
No	91.4% (±0.7)	91.9% (±0.8)	88.0% (±2.3)	93.7% (±2.7)	90.3% (±3.5)	*P* = 0.006
Yes	80.4% (±1.4)	82.7% (±1.5)	66.4% (±4.5)	73.5% (±6.6)	90.1% (±4.7)	*P* < 0.001
Stage						*P* < 0.001
0	99.3% (±0.5)	99.1% (±0.6)	100.0% (±0.0)	100.0% (±0.0)	100.0% (±0.0)	*P* = 0.874
I	97.5% (±0.6)	97.7% (±0.6)	98.8% (±1.2)	91.2% (±4.8)	97.4% (±2.6)	*P* = 0.285
II	89.8% (±1.0)	90.1% (±1.1)	84.6% (±3.6)	95.2% (±3.3)	93.2% (±3.8)	*P* = 0.012
III	70.5% (±2.4)	75.8% (±2.6)	48.5% (±6.7)	65.1% (±9.5)	67.1% (±13.5)	*P* < 0.001
IV	24.7% (±4.1)	21.7% (±4.6)	38.4% (±10.0)	28.6% (±17.1)	—	*P* = 0.460
Grade						*P* < 0.001
1	96.3% (±1.1)	97.7% (±1.0)	85.0% (±6.2)	100.0% (±0.0)	100% (±0.0)	*P* = 0.024
2	90.4% (±0.9)	91.1% (±1.0)	82.8% (±3.5)	91.8% (±3.9)	95.9% (±2.8)	*P* < 0.001
3	79.9% (±1.4)	80.9% (±1.6)	71.5% (±4.7)	73.8% (±6.8)	87.9% (±5.7)	*P* = 0.034
Unknown	88.6% (±1.6)	90.8% (±1.6)	84.7% (±4.5)	82.7% (±7.9)	70.6% (±11.1)	*P* = 0.338

**Table 5:** Cox regression multivariate analysis.

Prognostic factor	Hazard ratio	95% confidence interval	*P* value
Race^a^	1.386	1.185–1.621	*P* < 0.001
Age at diagnosis^b^	1.049	1.044–1.054	*P* < 0.001
Year of diagnosis^b^	0.983	0.971–0.994	*P* = 0.004
Lymph node dissection^c^	0.581	0.503–0.671	*P* < 0.001
T stage^d^	1.452	1.315–1.603	*P* < 0.001
Stage of disease^e^	1.203	1.114–1.300	*P* < 0.001
Grade^f^	1.237	1.134–1.349	*P* < 0.001

^a^Black versus non-Black.

^b^Continuous.

^c^Yes versus no.

^d^Metastatic disease versus 4 versus 3 versus 2 versus 1 versus in situ.

^e^IV versus III versus II versus I versus 0.

^f^3+ unknown versus 2 versus 1.
